# How Not to Be a Bioinformatician

**DOI:** 10.1186/1751-0473-7-3

**Published:** 2012-05-28

**Authors:** Manuel Corpas, Segun Fatumo, Reinhard Schneider

**Affiliations:** 1 Independent, Cambridge, UK; 2 Covenant University Nigeria, Department of Computer and Information Sciences, KM 10 Idiroko road, Ota, Nigeria; 3 Data Integration and Knowledge Management, European Molecular Biology Laboratory (EMBL), Meyerhofstr. 1, 69117, Heidelberg, Germany

## Abstract

Although published material exists about the skills required for a *successful* bioinformatics career, strangely enough no work to date has addressed the matter of how to excel at *not* being a bioinformatician. A set of basic guidelines and a code of conduct is hereby presented to re-address that imbalance for fellow-practitioners whose aim is to *not* to succeed in their chosen bioinformatics field. By scrupulously following these guidelines one can be sure to regress at a highly satisfactory rate.

## Introduction

The advent of fast 3D gaming PCs, the Internet and massive sequencing efforts have attracted hackers and failed wet-lab biologists to the bioinformatics field. To make matters worse, the looming prospect of massive lay-offs in the financial sector or the new hoped-for thrill to be working on things that matter [1] following a mid-life crisis, have all contributed to the resurgence of a new breed of a mutant, super-resistant bioinformatician species.

The resulting ecosystem of practitioners is an eclectic mixture of individuals, including a) those whose graphics card for video games would preferably be hammered out with algorithmic computations for the elucidation of the meaning of life, b) those submerged in the muddy waters of the ever increasing –omics subfields, and c) those fulfilling their role as annotation monkeys. As if that wasn’t enough, a myriad of new packages, formats and databases keep on mushrooming daily throughout the biomedical arena, surreptitiously embracing complexity just for the sake of it. Bioinformatics has evolved into the cheap form of Biology during cycles of funding shortages. Its tentacles now spread into pretty much every branch of life sciences. So much so that the ways of defining it are nearly as numerous as there are individuals in the field. After quite some time of wrestling with the idea of what Bioinformatics is, we decided that it would probably make more sense to define how *not* to become one of the troupe. So what follows is a compendium of 10 “sarcastic” guidelines that illustrate how a few months on the computer can save a few hours in the library (or on Google) [2].

1. **Stay low level at every level**. Develop your code by anecdote: avoid planning phases, requirement analysis exercises or any structure to your code. Stay away from object oriented programming. Build up your own little myriad of helper scripts. Do not document either inside or outside your code. Your coding style should only be understood by you. Make sure your software does not scale. Refuse to model or abstract and always choose the quick and dirty fix.

2. **Be open source without being open.** Error messages should never be provided. If error messages are provided, they should be utterly cryptic so as to convey as little information as possible to the end user [3]. If you create the application, make it difficult to build. Have plenty of hidden dependencies and bizarre variables. Don’t bother to debug or provide backwards compatibility. Ensure that your code is not portable, it only works in outdated operating systems and assume only you will use your application. Take for granted that everyone will be able to understand it.

3. **Make tools that make no sense to biologists.** The less they resemble any intelligible biological question the better. If you provide a help document, bombard scientists with abbreviations and provide as much unnecessary technical information as possible. The typical biologist hates mathematics, so use mathematical formulas extensively throughout the documentation. Integrate your workflows with as many irrelevant services as possible, so you’ll have greater the chances of a potential dead link.

4. **Do not provide a graphical user interface: command line is always more effective.** Force your end-users to use the command line. It helps if the parameter name does not relate to the intended action. For example, never use –o for specifying an output file; a “k” or “B” creates a more memorable impression. If you provide a graphical user interface, a) make sure there is no logic behind it, b) it is not intuitive to the user and c) support as few formats as you can, preferably html or text only. Forget HTTP-XML or SOAP. To make sure that the user experience is a nightmare, here are some guiding principles: 1) Provide thousands of menu options and pop up windows that make little or no sense. 2) Ask the user to make impossible decisions. 3) Change your interface/format whenever you feel like it, especially if many people and applications depend on it.

5. **Make sure the output of your application is unreadable, unparseable and does not comply to any known standards.** Just use plain ASCII text, or better still, provide your own obfuscated format. Do not use ontologies, XML, or any other inter-exchangeable standard. If you use XML, make sure that your data file is impossible to validate and that it does not follow any XML schema. You can also invent a new name for your gene if a) it doesn’t match any available identifier from reference databases and b) you don’t tell anyone about it.

6. **Be unreachable and isolated.** Configure your contact email to either bounce back or permanently set it to vacation. Miss key meetings or seminars where other colleagues may be presenting their seminal results and never, ever make any attempt at remembering their names or where they work. Reinvent the wheel. Do not keep up with the literature on current methods of research if you possibly can.

7. **Never maintain your databases, web services or any information that you may provide at any time.** Provide unstable data, unstable models and unstable services. Your ultimate goal in data curation should be to propagate as many errors as possible from one database to another, while still making sure that they sound realistic. Your curated data should only partially reflect the science of the papers you don’t read. When curating your data, make as many new categories as exceptions you find to your classifications. Forget about the final scientific question you are trying to answer and stay well away from convention.

8. **Blindly believe in the predictions given, P-values or statistics.** Select instances for your training set that you know will give you the answer you want. Produce arbitrary cut-offs on rank-ordered result lists. Absolute truth above, absolute falsehood below [2]. Always work with default parameters and never explore other options algorithms might provide. If you get a list of hits, only look at the first one. Do not believe in the mantra "rubbish in, rubbish out"; just make sure that your rubbish data doesn’t smell.

9. **Do not ever share your results and do not reuse.** Never discuss your results before your submission has been accepted in a lost conference proceeding. Consider that the work others are doing is probably a waste of time. Ignore whatever new algorithms and methods your colleagues have developed in the last two decades.

10. **Make your algorithm or analysis method irreproducible.** The less testing you carry out in your experiments, the more revolutionary results you should expect to get. When testing your algorithm, compare it against methods developed before the past decade: your performance levels will look much better. Include irrelevant variables in your equations and make them unnecessarily complex, so your reviewers will be very impressed by the complexity of the astonishing predictions you get.

## Conclusions

Here we have highlighted a series of disastrous practices in the bioinformatics field. We hope that readers will not only find them stimulating but useful. Embrace them fully and we guarantee their efficacy.

## Competing interests

The authors declare that they have no competing interest.

## Authors' contributions

MC, SF and RS conceived and wrote the paper. All authors have read and approved the final manuscript.
